# Electrophysiological Abnormalities in VLCAD Deficient hiPSC-Cardiomyocytes Do not Improve with Carnitine Supplementation

**DOI:** 10.3389/fphar.2020.616834

**Published:** 2021-01-12

**Authors:** Arie O. Verkerk, Suzan J. G. Knottnerus, Vincent Portero, Jeannette C. Bleeker, Sacha Ferdinandusse, Kaomei Guan, Lodewijk IJlst, Gepke Visser, Ronald J. A. Wanders, Frits A. Wijburg, Connie R. Bezzina, Isabella Mengarelli, Riekelt H. Houtkooper

**Affiliations:** ^1^ Department of Clinical and Experimental Cardiology, Heart Center, Amsterdam Cardiovascular Sciences, Amsterdam UMC, University of Amsterdam, Amsterdam, Netherlands; ^2^ Department of Medical Biology, Amsterdam Cardiovascular Sciences, Amsterdam UMC, University of Amsterdam, Amsterdam, Netherlands; ^3^ Laboratory Genetic Metabolic Diseases, Amsterdam UMC, University of Amsterdam, Amsterdam Gastroenterology and Metabolism, Amsterdam Cardiovascular Sciences, Amsterdam, Netherlands; ^4^ Department of Pediatric Metabolic Diseases, Wilhelmina Children’s Hospital, University Medical Center Utrecht, Utrecht, Netherlands; ^5^ Institute of Pharmacology and Toxicology, Technische Universität Dresden, Dresden, Germany; ^6^ Department of Pediatric Metabolic Diseases, Emma Children's Hospital, Amsterdam UMC, University of Amsterdam, Amsterdam, Netherlands

**Keywords:** very long-chain acyl-CoA dehydrogenase, arrhythmia < cardiovascular, acylcarnitines, action potential, human induced pluripotent stem cell derived cardiomyocytes, patients, treatment, carnitine

## Abstract

Patients with a deficiency in very long-chain acyl-CoA dehydrogenase (VLCAD), an enzyme that is involved in the mitochondrial beta-oxidation of long-chain fatty acids, are at risk for developing cardiac arrhythmias. In human induced pluripotent stem cell derived cardiomyocytes (hiPSC-CMs), VLCAD deficiency (VLCADD) results in a series of abnormalities, including: 1) accumulation of long-chain acylcarnitines, 2) action potential shortening, 3) higher systolic and diastolic intracellular Ca^2+^ concentrations, and 4) development of delayed afterdepolarizations. In the fatty acid oxidation process, carnitine is required for bidirectional transport of acyl groups across the mitochondrial membrane. Supplementation has been suggested as potential therapeutic approach in VLCADD, but its benefits are debated. Here, we studied the effects of carnitine supplementation on the long-chain acylcarnitine levels and performed electrophysiological analyses in VLCADD patient-derived hiPSC-CMs with a *ACADVL* gene mutation (p.Val283Ala/p.Glu381del). Under standard culture conditions, VLCADD hiPSC-CMs showed high concentrations of long-chain acylcarnitines, short action potentials, and high delayed afterdepolarizations occurrence. Incubation of the hiPSC-CMs with 400 µM L-carnitine for 48 h led to increased long-chain acylcarnitine levels both in medium and cells. In addition, carnitine supplementation neither restored abnormal action potential parameters nor the increased occurrence of delayed afterdepolarizations in VLCADD hiPSC-CMs. We conclude that long-chain acylcarnitine accumulation and electrophysiological abnormalities in VLCADD hiPSC-CMs are not normalized by carnitine supplementation, indicating that this treatment is unlikely to be beneficial against cardiac arrhythmias in VLCADD patients.

## Introduction

Patients with a deficiency in very long-chain acyl-CoA dehydrogenase (VLCAD; EC 1.3.99.3), the enzyme catalyzing the first step of the mitochondrial beta-oxidation of long-chain fatty acids (Houten and Wanders, 2010; Knottnerus et al., 2018), are at risk for developing liver, skeletal, and heart muscle dysfunction (Ribas and Vargas, 2020; Wanders et al., 2020), including cardiac arrhythmias (Bonnet et al., 1999). Traditionally patients with VLCAD deficiency (VLCADD; OMIM 609575) are treated with dietary restriction of long-chain triglycerides, supplementation of medium-chain triglycerides, and prevention of catabolic state (Yamada and Taketani, 2019). Carnitine supplementation had also been proposed for patients with VLCADD in order to treat secondary carnitine deficiency and to increase the transport of acyl compounds out of the mitochondria (Treem et al., 1991; Winter, 2003).

Carnitine is required for the transport of activated long-chain fatty acids into the mitochondrial matrix, by formation of long-chain acylcarnitines (LCAC) from acyl-CoA esters, and is therefore essential for fatty acid oxidation (Houten and Wanders, 2010). In humans, carnitine is mainly derived from the diet, but can also be synthesized by the liver, kidney and brain (Vaz and Wanders, 2002; Reuter and Evans, 2012; Wanders et al., 2020). Tissues like skeletal muscle and the heart acquire carnitine from the circulation. Its indispensable role in metabolism is illustrated by patients who suffer from primary carnitine deficiency (OMIM 212140) due to excessive urinary carnitine wasting (Longo et al., 2006). So far, the evidence for a beneficial effect of carnitine supplementation in VLCADD patients is not conclusive (Nasser et al., 2012; (Spiekerkoetter et al., 2010; Ribas and Vargas, 2020). A major concern may be the potential increased formation in LCACs (Spiekerkoetter et al., 2009; Ribas and Vargas, 2020), especially since LCACs are associated with the development of cardiac arrhythmia (Corr et al., 1989). On the other hand, extremely low free carnitine levels, as seen in patients with primary carnitine deficiency, are also linked to abnormalities in electrical morphology of the heart (Roussel et al., 2016). Therefore, more insight on effects and safety of carnitine supplementation on electrophysiological abnormalities for VLCADD treatment is needed.

So far, the cardiac effects of carnitine supplementation have been studied in mice. In VLCADD murine models, it was found that supplementation of carnitine can indeed lead to a more pronounced accumulation of LCACs (Liebig et al., 2006; Primassin et al., 2008) without replenishment of free carnitine in cardiac tissue (Liebig et al., 2006). This is in contrast with the findings in a long-chain acyl-CoA dehydrogenase (LCAD) knock out (KO) murine model, in which carnitine supplementation did not lead to increased long-chain acylcarnitine levels and even normalized myocardial triglycerides (Bakermans et al., 2013). However, mice are considerably different from humans with respect to fatty acid oxidation as well as carnitine metabolism (Chegary et al., 2009), and therefore it is uncertain whether findings in these murine models can be directly extrapolated to the human situation and be applied to formulation of a human therapy. Very recently, we generated a human-based cardiac VLCADD model by generation of human induced pluripotent stem cells (hiPSCs) from patients carrying mutations in the VLCAD-encoding *ACADVL* gene and differentiated them into cardiomyocytes (hiPSC-CMs) (Knottnerus et al., 2020). We found that VLCAD deficiency in this hiPSC-CMs model results in accumulation of LCACs, higher systolic and diastolic intracellular Ca^2+^ (Ca^2+^_i_) concentrations, action potential (AP) shortening, and development of delayed afterdepolarizations (DADs). Furthermore, we found that pre-incubation of VLCADD hiPSC-CMs with either resveratrol or etomoxir–compounds that give rise to an enhanced mitochondrial biogenesis or inhibit fatty acid transport into the mitochondria, respectively (Lopaschuk et al., 2010; Houtkooper et al., 2012)– led to the normalization of the acylcarnitine levels and restored electrophysiological and Ca^2+^_i_ abnormalities (Knottnerus et al., 2020). Importantly, these findings suggest that treatment with such compounds may be beneficial for VLCADD patients. In addition, these findings also indicate that our hiPSC-CM model is suitable for drug discovery studies of this metabolic disorder. In the present study, we used the VLCADD hiPSC-CM model to study the effects of carnitine supplementation on the LCAC profile and cellular electrophysiology.

## Materials and Methods

### Human Induced Pluripotent Stem Cell Derived Cardiomyocytes Generation

hiPSC-CMs were generated from the hiPSC line, iVLCADD1, which was derived from skin fibroblasts of a VLCADD-affected woman with mutations (p.Val283Ala/p.Glu381del) in the *ACADVL* gene (Knottnerus et al., 2020). Differentiation of the hiPSCs into hiPSC-CMs was performed in RPMI 1,640 medium supplemented with B27 (Gibco), initiated with CHIR99021, Activin A and BMP4, followed by Wnt-pathway inhibition by IWP4, differentiation proceeded in presence of RPMI medium supplemented with B27 and insulin until day 30 as we have previously described in detail (Knottnerus et al., 2020). Next, we performed a metabolic selection-based enrichment for hiPSC-CMs by applying glucose-depleted RPMI medium containing 4 mM lactate for six days, thereby removing a large proportion of non-cardiomyocytes (Tohyama et al., 2013). During the whole process, no serum was supplied to the culture medium. Finally, 36 days after the start of the differentiation process, hiPSC-CMs were dissociated to single cells using a two-step procedure starting with 5× TrypLE Select Enzyme (Gibco) to detach the cells from culture flasks, followed by a combination of liberase (Roche Chemicals) and Elastase (Serva) to obtain single cells (Knottnerus et al., 2020). The cells were seeded on matrigel-coated glass coverslips and cultured for 10 days in RPMI medium supplemented with 2% B27, 50 U/ml penicillin, and 50 µg/ml streptomycin and 48 h prior to measurement 400 µM L-carnitine (C0158, Sigma-Aldrich) was added to the culture medium in paired experiments. hiPSC-CMs generated from a control hiPSC line, iCTRL (Dudek et al., 2013), were used as control and were cultured in the absence of carnitine.

### Acylcarnitine Profiling

The intracellular acylcarnitine profile of the hiPSC-CMs was assessed after 30 days of differentiation as described previously (Knottnerus et al., 2020). In short, hiPSC-CMs from the iCTRL and VLCADD1 line, here named iCTRL-CMs and iVLACDD1-CMs respectively, were harvested with trypsin and cell pellets or 50 µL of culture medium were extracted in 500 µL acetonitrile with 10 µL internal standards (^2^H_3_-C3-carnitine, ^2^H_3_-C8 carnitine, ^2^H_3_-C16 carnitine) and measured with high performance liquid chromatography mass spectrometry on a Q-ExactiveTM mass spectrometer (Thermo Scientific). Data was normalized to level of protein per well, determined using bicinchonic acid (BCA) assay with human serum albumin (HSA) in 0.4 mmol/L NaOH used as a standard (Knottnerus, et al., 2020).

### Patch-Clamp Measurements

#### Data Acquisition

APs and afterdepolarizations were recorded at 36 ± 0.2°C using the perforated patch-clamp technique and an Axopatch 200B amplifier (Molecular Devices, Sunnyvale, CA, United States). Data acquisition, voltage control, and analysis were accomplished using custom software. Signals were low-pass filtered with a cut-off frequency of 5 kHz and digitized at 40 and 3 kHz for APs and DADs, respectively. Cell membrane capacitance (C_m_, in pF) was estimated by dividing the time constant of the decay of the capacitive transient in response to 5 mV hyperpolarizing voltage clamp steps from −40 mV by the series resistance. C_m_ of hiPSC-CMs was 23.9 ± 1.3 pF (mean ± SEM, n = 41), and did not differed significantly between iCTRL-CMs, untreated iVLCADD1-CMs, and carnitine-treated iVLCADD1-CMs. Patch pipettes with a resistance of 2–3 MΩ were pulled from borosilicate glass (Harvard Apparatus) and filled with solution containing (in mM): 125 K-gluconate, 20 KCl, 5 NaCl, 0.44 Amphotericin-B, 10 HEPES; pH set to 7.2 (KOH). Cells were superfused with modified Tyrode’s solution containing (in mM): 140 NaCl, 5.4 KCl, 1.8 CaCl_2_, 1.0 MgCl_2_, 5.5 D-glucose, 5 HEPES; pH set to 7.4 (NaOH). All potentials were corrected for the estimated liquid junction potential of −15 mV (Barry and Lynch, 1991).

#### Action Potential Recordings

hiPSC-CMs offer a great human-based model for cardiac disease modeling (Hoekstra et al., 2012), and drug discovery and cardiotoxicity screenings (Mordwinkin et al., 2013), but a major limitation for electrophysiological studies is that they have a small or even complete lack of the inward rectifying K^+^ current (I_K1_) (Veerman et al., 2015). I_K1_ plays a major role in maintaining a stable resting membrane potential in cardiomyocytes (Nerbonne and Kass, 2005), and consequent to its absence, hiPSC-CMs have a depolarized maximal diastolic potential and are frequently spontaneously active. To overcome this limitation, we injected an in silico I_K1_ with kinetics of Kir2.1 channels and a 2 pA/pF current density through dynamic clamp, as previously described in detail (Meijer van Putten et al., 2015). The dynamic clamp injected I_K1_ resulted in quiescent hiPSC-CMs and we elicited APs at 0.2–4 Hz by 3 ms, ≈1.2× threshold current pulses through the patch pipette. Susceptibility to DADs was tested by applying a 3 Hz pacing episode (10 s) followed by an 8 s pause. After the pause, a single AP was evoked to test the inducibility of early afterdepolarizations (EADs). DADs were defined as depolarizations that occurred after the fast pacing and which were larger than 1 mV. EADs were defined as oscillations in membrane potential which interrupt or retard repolarization of the AP were defined as EADs accordingly to the definition of Cranefield (1977). The AP parameters analyzed were maximum diastolic potential (MDP, in mV), maximal AP amplitude (APA, in mV), maximum upstroke velocity (V_max_, in V/s) AP duration at 20, 50, and 90% repolarization (APD_20_, APD_50_, and APD_90_, respectively, in ms). Parameters from 10 consecutive APs were averaged and number of DADs and EADs was counted and averaged over five fast pacing recording traces.

### Statistical Analysis

Statistical analysis was carried out with SigmaStat 3.5 software (Systat Software, Inc., San Jose, CA, United States). Normality and equal variance assumptions were tested with the Kolmogorov-Smirnov and the Levene median test, respectively. Group comparisons were performed with unpaired t-test, One-Way ANOVA followed by Holm-Sidak post hoc test, Two-Way Repeated Measures ANOVA followed by pairwise comparison using the Student–Newman–Keuls test or Fisher Exact Test with Freeman-Halton extension.

In case of non-normally distributed parameters, Kruskal-Wallis tests followed by pairwise comparisons with Dunn’s methods were applied. Data are presented as mean ± SEM or as boxplots, in which boxes represent lower quartile, median and upper quartile, and whiskers 1.5× interquartile range. *p* < 0.05 was considered statistically significant.

## Results

### Long-Chain Acylcarnitine Concentrations

**FIGURE 1:**
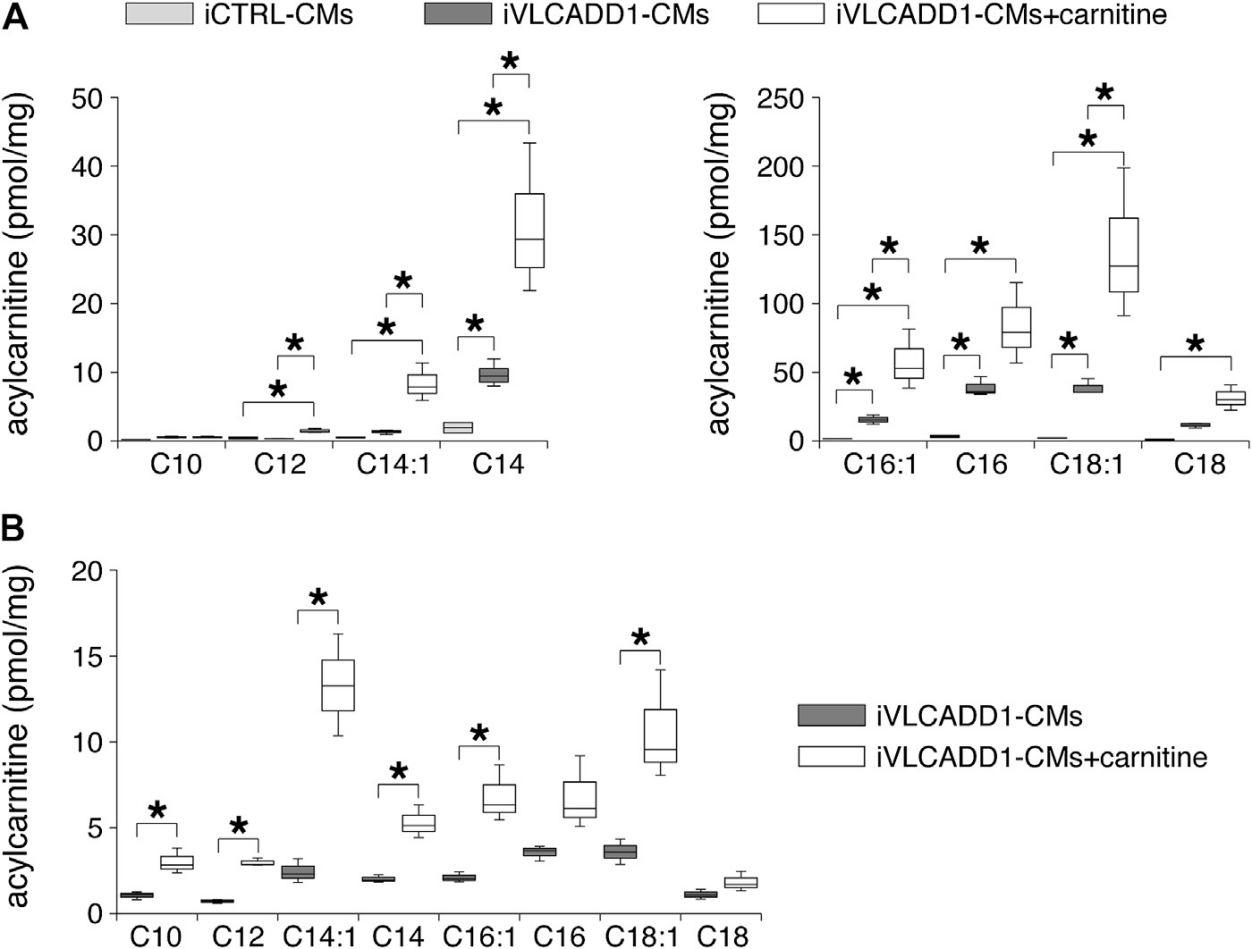
Box plots of acylcarnitine profiles in cell pellets and culture medium. **(A)** Box plots of acylcarnitine profiles in cell pellets of iCTRL-CMs and iVLCADD1-CMs cultured in absence or in presence of 400 µM carnitine for 48 h. Asterisks indicate significant differences between groups (One-Way ANOVA followed by Holm-Sidak post hoc test or Kruskal-Wallis tests followed by pairwise comparisons with Dunn’s methods). Data from three differentiations obtained from cell pellets 30 days after differentiation. **(B)** Box plots of acylcarnitine profiles measured in culture medium of VLCADD1-CMs cultured under standard culture conditions or in presence of 400 µM carnitine for 48 h. Asterisks indicate significant differences between groups (Unpaired t-test; data from three differentiations measured 30 days after differentiation.

First, we measured the acylcarnitine profiles in iCTRL-CMs and iVLCADD1-CMs cultured under standard culture conditions and in the presence of 400 µM carnitine for 48 h. Compared to iCTRL-CMs, LCAC concentrations were higher in iVLCADD1-CMs cultured in the absence of carnitine (Figure 1A). Culturing the iVLCADD1-CMs in the presence of carnitine resulted even in a further increase (Figure 1A). For example, C18:1-acylcarnitine increased from 38.8 ± 4.0 pmol/mg (three replicates) in iVLCADD1-CMs cultured in standard medium without additional carnitine to 138.5 ± 38.9 pmol/mg (three replicates) in iVLCADD1-CMs cultured with carnitine. In iVLCADD1-CMs, carnitine also resulted in a significant increase in LCACs levels in the culture medium with most prominent effects on C14:1-acylcarnitine (Figure 1B). This indicates that the hiPSC-CMs are able to excrete the accumulating LCACs.

### Carnitines Incubation Does Not Affect Action Potentials

**FIGURE 2:**
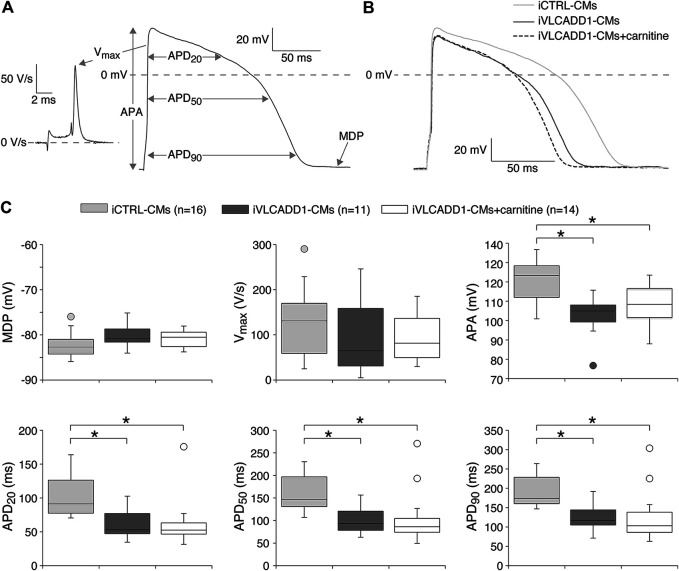
Action potential (AP) characteristics at 1 Hz of single iCTRL-CMs and iVLCADD1-CMs cultured in absence or presence of carnitine. **(A)** Illustration of the analyzed AP parameters, i.e., maximum diastolic potential (MDP), AP amplitude (APA), maximum upstroke velocity (V_max_), and AP duration at 20, 50, and 90% repolarization (APD_20_, APD_50_, and APD_90_, respectively). **(B)** Typical APs of iCTRL-CMs and iVLCADD1-CMs cultured in absence or presence of carnitine. **(C)** Box plots of the APs characteristics. Asterisks indicate significant differences between groups (One-Way ANOVA followed by Holm-Sidak post hoc test or Kruskal-Wallis tests followed by pairwise comparisons with Dunn’s methods). iCTRL-CM and iVLCADD1-CM data are from five to four differentiations, respectively, and were measured 46 and 47 days after differentiation.

Next, we assessed the AP parameters of the iCTRL-CMs and compared them to those of the iVLCADD1-CMs cultured in the absence or presence of carnitine. The AP parameters were analyzed as depicted in Figure 2A. Figure 2B shows a typical AP at 1 Hz stimulation of an iCTRL-CMs as well as typical 1 Hz stimulated APs recorded from an iVLCADD1-CMs cultured in the absence or presence of carnitine. Average AP parameters obtained from a total of 16 iCTRL-CMs, 11 iVLCADD1-CMs cultured in standard conditions, and 14 iVLCADD1-CMs cultured in the presence of carnitine are summarized in Figure 2C. The average MDP was around −80 mV and the V_max_ was between 50 and 150 V/s, but both MDP and V_max_ did not differ significantly between the three hiPSC-CMs groups. In all cell lines, APs overshot the 0 mV level, but the APA was significantly lower in the iVLCADD1-CMs in the absence as well as in the presence of carnitine compared to the iCTRL-CMs. iCTRL-CMs had an APA of 120 ± 2.7 mV (n = 16), while the iVLCADD1-CMs lines showed an APA of 99 ± 3.9 mV (n = 11) and 102 ± 3.4 mV (n = 14) in the absence and presence of carnitine, respectively. APs of both iVLCADD1-CMs groups repolarized earlier and faster than iCTRL-CMs (Figure 2A), resulting in a significantly shorter APD_20_, APD_50_, and APD_90_. For example, APD_90_ was 190 ± 9.7 mV (n = 16) in iCTRL-CMs and 127 ± 12.2 mV (n = 11) and 126 ± 21.0 mV (n = 14) in the iVLCADD1-CMs in absence and presence of carnitine. Neither APA nor the APDs differ significantly between iVLCADD1-CMs cultured in the presence of 400 µM carnitine and iVLCADD1-CMs cultured under standard conditions.

**FIGURE 3:**
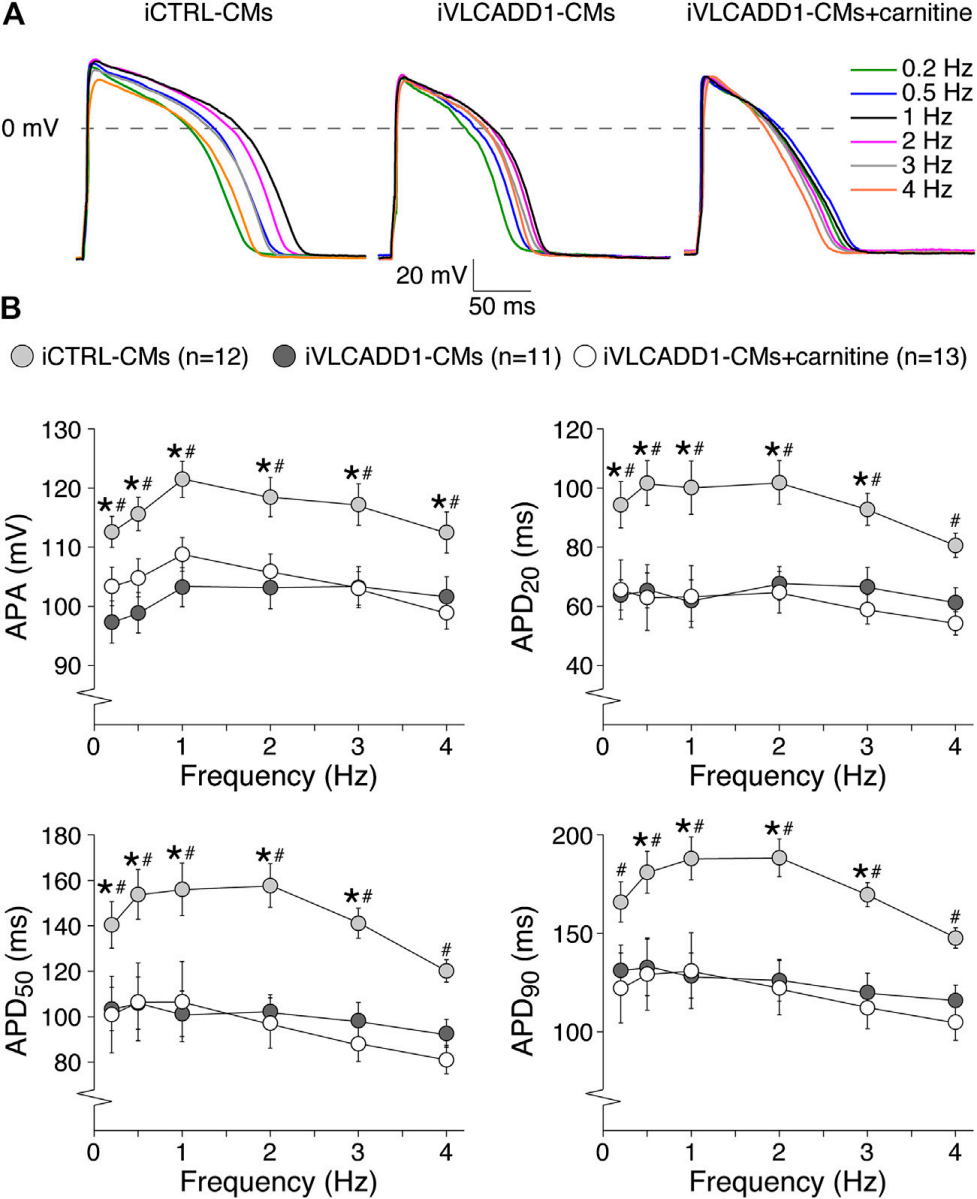
AP parameters at 0.2–4 Hz pacing of iCTRL-CMs and iVLCADD1-CMs cultured in absence or presence of carnitine. **(A)** Typical APs of iCTRL-CMs and iVLCADD1-CMs cultured in absence or presence of carnitine stimulated at 0.2, 0.5, 1, 2, 3, and 4 Hz. **(B)** APA, APD_20_, APD_50_, and APD_90_ at 0.2–4 Hz. Asterisks indicate significant differences between groups (Two-way Repeated Measures ANOVA). iCTRL-CM and iVLCADD1-CM data are from five to four differentiations, respectively, and were measured 46 and 47 days after differentiation.

**FIGURE 4:**
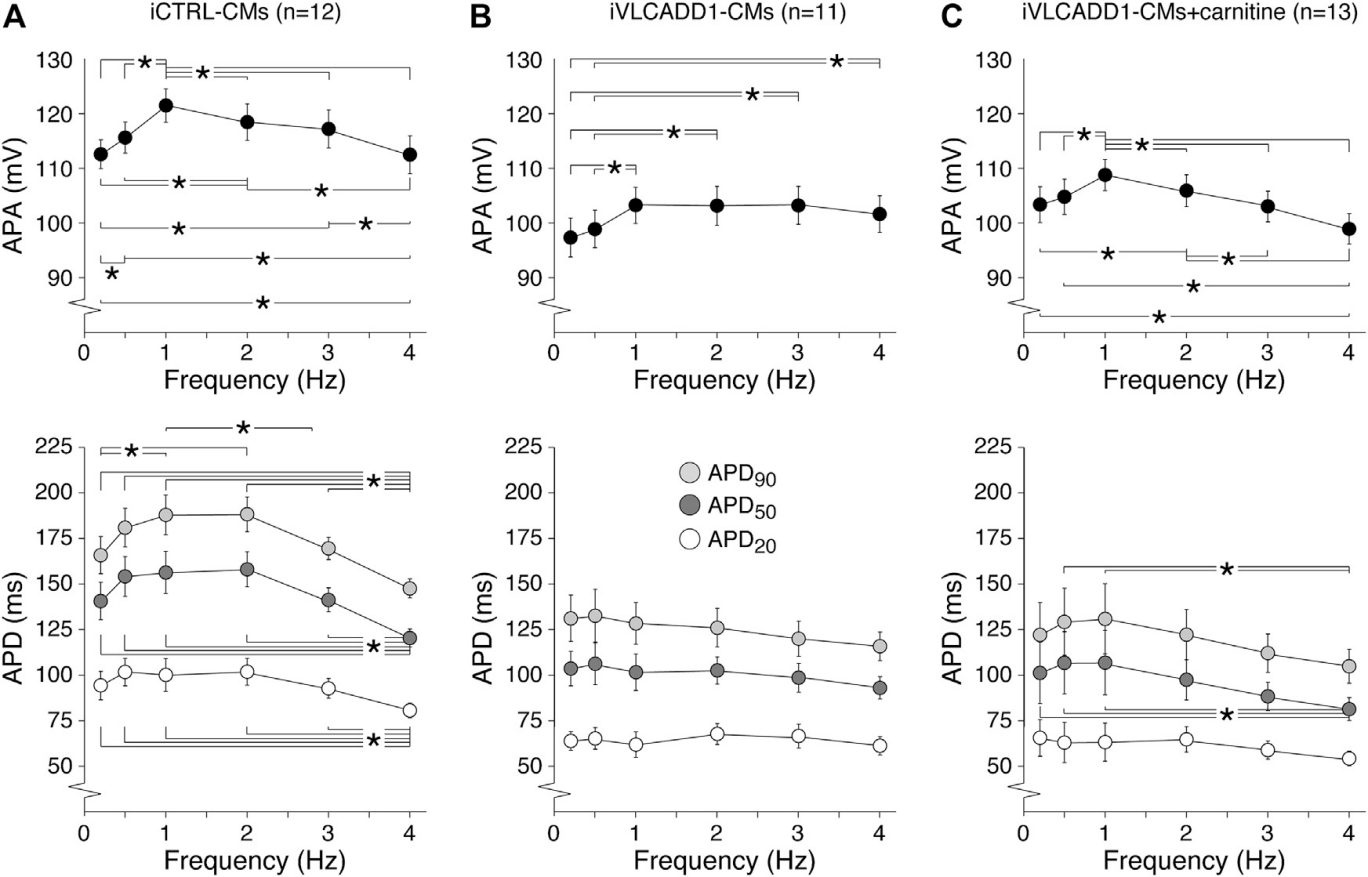
**(A–C)** Frequency dependence of APA, APD_20_, APD_50_, and APD_90_ in iCTRL-CMs **(A)** and iVLCADD1-CMs cultured in absence **(B)** or presence of carnitine **(C)**. Asterisks indicate significant differences between stimulus frequencies (Two-way Repeated Measures ANOVA). iCTRL-CM and iVLCADD1-CM data are from five to four differentiations, respectively, and were measured 46 and 47 days after differentiation.

Cardiac APs show a frequency dependence in morphology (Carmeliet, 2004) and the iVLCADD1-CM electrical phenotype as well as potential effects of carnitine may be frequency-related. For this reason, we studied the AP characteristics at 0.2–4 Hz stimulation. Figure 3A shows typical APs and Figure 3B summarizes the average AP parameters that were significantly different between iCTRL-CMs and iVLCADD1-CMs at 1 Hz. APA, APD_20_, APD_50_, and APD_90_ of iCTRL-CMs were significantly different from iVLCADD1-CMs cultured without and in the presence of carnitine at all frequencies, with exception of the APDs at 4 Hz in absence of carnitine (Figure 3B). As illustrated in Figure 3, the APs of iCTRL-CMs shows a clear frequency dependency with a decrease in APA and APDs upon both slower and faster pacing frequencies compared to the 1 Hz pacing frequency. This is depicted in more detail in Figure 4A, in which the iCTRL-CMs data presented in Figure 3B are replotted, but now with asterisks which indicate the significant differences between stimulus frequencies. In the VLCADD1 lines on the other hand, frequency dependence, especially those of the APDs, was less prominent as can be seen from the limited number of significant differences (Figures 4B,C). Thus, in iVLCADD1-CMs, APA and APDs are decreased at all frequencies compared to iCTRL-CMs, whereas the frequency dependence of AP parameters is hardly present in iVLCADD1-CMs. In addition, these data demonstrate that carnitine does not prevent these effects.

### Carnitines Incubation Does Not Affect the Occurrence of Afterdepolarizations

**FIGURE 5:**
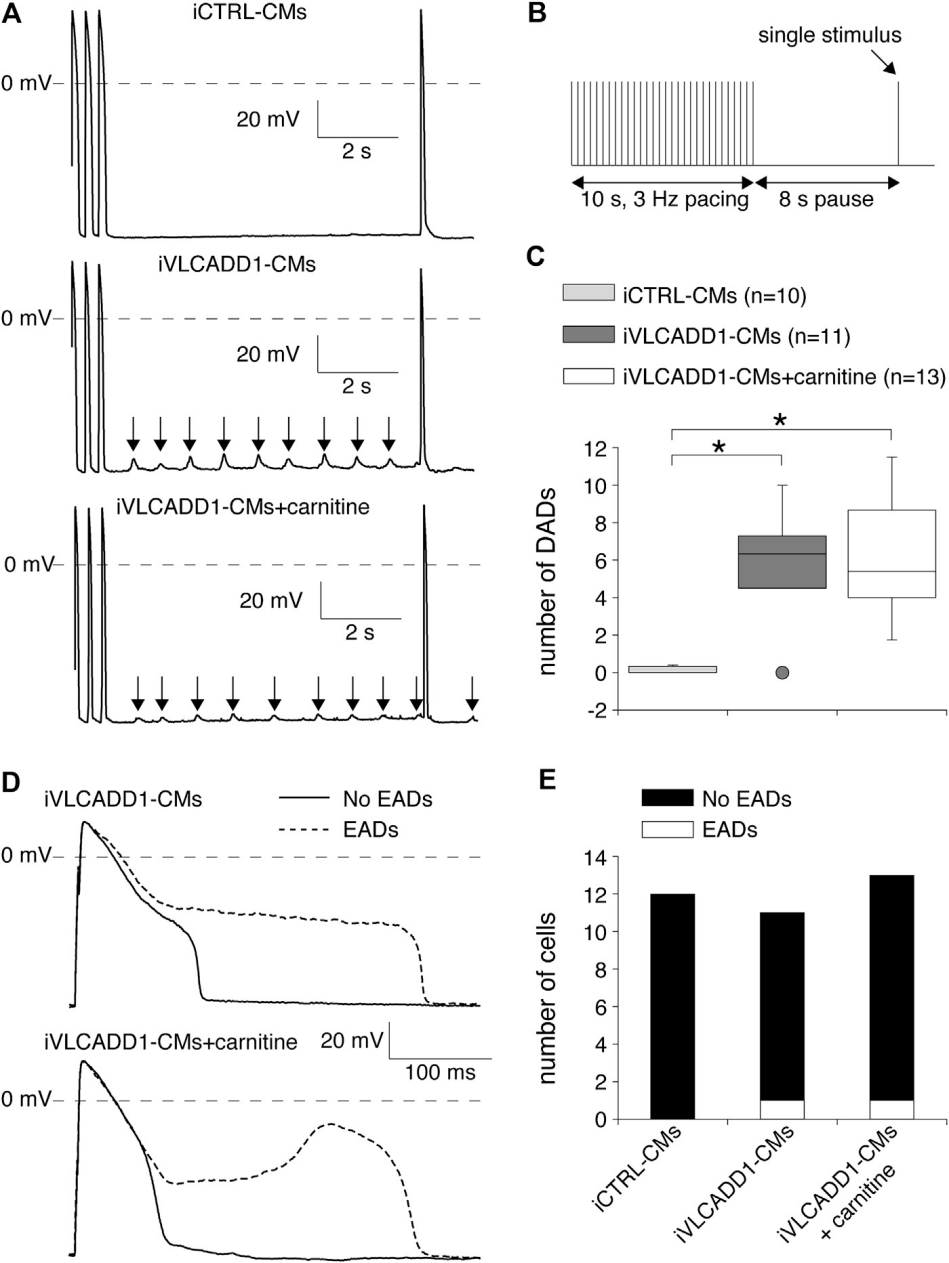
Delayed and early afterdepolarization (DAD and EAD, respectively) inducibility of iCTRL-CMs and/or iVLCADD1-CMs cultured in absence or presence of carnitine. **(A)** Typical DAD examples of an iCTRL-CM and iVLCADD1-CMs cultured in absence or presence of carnitine. The arrows indicate the DADs. **(B)** Illustration of the protocol to induce DADs. **(C)** Average occurrence of DADs. **(D)** APs at 0.2 Hz pacing of an iVLCADD1-CM cultured in absence and an iVLCADD1-CM cultured in presence of carnitine. EADs were only present once every three to five APs and the APs exhibiting an EAD are indicated by dashed lines. **(E)** EADs occurrence at 0.2 Hz in iCTRL-CMs and iVLCADD1-CMs cultured in absence or presence of carnitine. Asterisks indicate significant differences between groups (Kruskal-Wallis test followed by pairwise comparisons with Dunn’s methods). iCTRL-CM and iVLCADD1-CM data are from five to four differentiations, respectively, and were measured 46 and 47 days after differentiation.

We also assessed the effects of carnitine on the occurrence of DADs and EADs, both important cellular electrophysiological triggers for cardiac arrhythmias (Hoffman and Rosen, 1981; Wit, 2018). Susceptibility to DADs was tested by applying a fast, 3-Hz pacing episode (10-s) followed by an 8-s pause (Figure 5B). After the pause, we evoked a single AP (Figure 5B) to test the inducibility of EADs. In addition, we counted the occurrence of EADs during continuous 0.2 Hz stimulation. The results of DADs and EADS are shown in Figures 5A,B, respectively.

As illustrated in the typical examples of Figures 5A, fast pacing resulted in DADs (indicated with arrows) in the post-pacing pause in both iVLCADD1-CMs groups and not in the iCTRL-CMs. Figure 5C summarizes the occurrence of DADs for a total of 10 iCTRL-CMs, 11 iVLCADD1-CMs cultured in the absence, and 13 iVLCADD1-CMs cultured in the presence of carnitine. As already observed in the typical examples of Figure 5A, and confirming our previous findings (Knottnerus et al., 2020), there is a significant increase of DADs in the iVLCADD1-CMs lines (Figure 5C). However, the number of DADs was not significantly different between the iVLCADD1-CMs cultured in absence and presence of carnitine. EADs were never observed during the single AP evoked after the 8-s pause. In addition to the experiments presented in Figure 5A, we re-analyzed the experiments in which we paced the hiPSC-CMs continuously at a slow 0.2 Hz stimulus frequency for the occurrence of EADs. The data, obtained from a total of 12 iCTRL-CMs, 11 iVLCADD1-CMs cultured in standard conditions, and 13 iVLCADD1-CMs cultured in the presence of carnitine, further demonstrates that the presence of EADs was rare (Figures 5D,E). In iVLCADD1-CMs cultured without as well as in iVLCADD1-CMs cultured in the presence of carnitine, we only observed EADs in one cell (Figures 5D,E) and the EADs occurred once every three to five APs. No EADs were ever observed in the iCTRL-CMs. Thus, these experiments demonstrate a high incidence of DADs in iVLCADD1-CMs, but highlight the lack of protective effect of carnitine supplementation in afterdepolarization occurrence.

## Discussion

In recent years, supplementation of L-carnitine to patients with a lcFAO disorder has remained a topic of debate (Spiekerkoetter et al., 2010; Pena et al., 2016; Yamada and Taketani, 2019). The main argument against L-carnitine supplementation is the suspicion that high levels of intrinsic and/or circulating LCACs may cause cardiac arrhythmias (Corr et al., 1989). Here, we studied the effects of L-carnitine supplementation in hiPSC-CMs generated from a VLCADD patient. We observed an increase in intracellular levels of LCACs, as well as an increase in excreted LCACs. There was neither an improvement in AP parameters nor in the occurrence of afterdepolarizations after L-carnitine supplementation. We conclude that LCACs accumulation and electrophysiological abnormalities in VLCADD hiPSC-CMs are not restored by carnitine supplementation, which suggests that carnitine treatment is not beneficial against cardiac arrhythmias in VLCADD patients.

iVLCADD1-CMs cultured under standard conditions exhibit a lower APA and shorter AP at a wide range of stimulus frequencies compared to iCTRL-CMs (Figures 2, 3). The exact mechanism is unknown, but is likely related to the LCAC accumulation (Figure 1A) and the consequent L-type Ca^2+^ current (I_Ca,L_) decrease (Wu and Corr, 1992) and increased systolic Ca^2+^_i_ concentrations resulting in enhanced Ca^2+^-induced I_Ca,L_ inactivation and increased K^+^ currents (Knottnerus et al., 2020). Indeed, reducing LCAC concentrations with either resveratrol or etomoxir restored APA and APDs in VLCADD hiPSC-CMs (Knottnerus et al., 2020). This pharmacological rescue of the metabolic and electrical phenotypes further indicates that these electrical abnormalities were not due to different genetic backgrounds in our used cell lines. The latter is also supported by experiments with a second control hiPSC line (for details, see Veerman et al., 2016), which show almost identical AP parameters (and the absence of DADs and EADs) as the presently used iCTRL-CMs (data not shown). While the iCTRL-CMs showed a clear frequency dependency of APA and APDs, this was less pronounced in the standard cultured iVLCADD1-CMs (Figure 4). The APA and APD decrease in iCTRL-CMs at faster stimulus frequencies is likely due to a combination of incomplete recovery of I_Ca,L_ and accumulation of repolarizing delayed rectifier K^+^ currents (Carmeliet, 2004). The AP shortening at slow stimulus frequencies in iCTRL-CMs, on the other hand, is likely due the repolarizing transient outward K^+^ current, I_to1_. In hiPSC-CMs, I_to1_ has an extremely slow recovery from inactivation (Cordeiro et al., 2013) and consequently plays only a role in AP repolarizing at low stimulus frequencies (Ma et al., 2018). The loss of frequency dependency of APA and APDs observed in iVLCADD1-CMs may be related to the already above mentioned decrease in I_Ca,L_ function and the obvious fast AP repolarization in iVLCADD1-CMs, which can mask subtle changes in net currents (Gaur et al., 2020). We found a high incidence of DADs in iVLCADD1-CMs cultured under standard conditions (Figures 5A,C), consistent with our previous study (Knottnerus et al., 2020). In human CMs, DADs are due to Ca^2+^_i_ overload-induced spontaneous Ca^2+^ release from the sarcoplasmic reticulum (SR), which activates an inward current carried by the Na^+^-Ca^2+^ exchanger (Verkerk et al., 2001). Ca^2+^_i_ overload was previously observed in hiPSC-CMs from VLCADD patients (Knottnerus et al., 2020). EADs in iCTRL-CMs were absent and rare in iVLCADD-CMs (Figures 5D,E). EADs can be divided in the so-called phase 2 and late-phase 3 EADs which are supposed to be due to different mechanisms (Qu et al., 2013). Phase 2 EADs are caused by re-activation of I_Ca,L_ which may occur during prolonged APs, while late-phase 3 EADs are likely due to the Na^+^-Ca^2+^ exchange current when the AP is substantially shortened such that the Ca^2+^ transient outlast the AP repolarization (Qu et al., 2013). It is tempting to define the observed EADs as late-phase 3 EADs based on the observed AP shortening (Figure 2) and previously observed increased systolic Ca^2+^_i_ (Knottnerus et al., 2020) which may result in a larger Na^+^-Ca^2+^ exchange current, but further studies are required to address this issue in detail.

The electrophysiological properties of the iVLCADD1-CMs cultured for 48 h in the presence of carnitine supplementation were not different from iVLCADD1-CMs cultured under standard conditions (Figures 2–5), demonstrating that the lack of effect of carnitine in restoring electrophysiological abnormalities. Supplementation of carnitine for 48 h was sufficient to increase the LCAC concentrations (Figure 1A), indicating that the absence of electrophysiological effects is not related to the length of the exposure time to the drugs in this particular hiPSC-CM model. In addition, we have previously observed that 48 h of drug treatment is sufficient to affect mitochondrial function in our iVLCADD1-CMs model, therefore indicating that this hiPSC-CMs model is useful for metabolic disorder drug studies (Knottnerus et al., 2020), even though hiPSC-CMs may have a less developed mitochondrial system for FAO flux compared to adult cardiomyocytes due to their immaturity (Chen et al., 2016). While the carnitine treatment increased the LCAC concentrations, electrophysiological abnormalities were not aggravated. This suggests that there is not a simple linear relationship between AP shortening and incidence of DADs and the LCACs. In addition, it indicates that carnitine treatment can be used for other, non-cardiac VLCADD-induced dysfunctions without increasing the risk for cardiac arrhythmias.

Secondary carnitine deficiency may occur in patients with VLCADD as a result of acylcarnitine formation from high levels of non-degraded acyl-CoAs. The pathophysiological relevance of carnitine depletion for the signs and symptoms observed in this disorder is not clear. Still, carnitine supplementation is often part of the treatment of VLCADD, especially when low (plasma) carnitine concentrations are found in patients (Pena et al., 2016). Evaluation of the efficacy of this intervention is very complex, due to the low numbers of patients and heterogeneous phenotypic presentations. In addition, acylcarnitine profiles measured in plasma do not correlate well with profiles measured in tissue (muscle tissue or white adipose tissue) (Schooneman et al., 2014). We found in iVLCADD1-CMs an increased LCAC accumulation as well as an increased LCAC secretion after carnitine supplementation. So far, there is limited knowledge on the cellular export mechanisms of acylcarnitines (Kim et al., 2017), but the increased LCAC accumulation induced by carnitine supplementation is in agreement with findings in mouse models for VLCADD (Liebig et al., 2006; Primassin et al., 2008; Bakermans et al., 2013). Bakermans and colleagues (Bakermans et al., 2013), however, only observed an increase of C16 and C18:1-carnitine whereas C14:1 levels were not affected in bloodspots after carnitine supplementation.

The heart lacks enzymes for carnitine synthesis and is therefore dependent on carnitine import from the circulation via the sodium dependent organic cation transporter (OCTN2). In a recent study, it was shown that haploinsufficiency of OCTN2 in lcFAO disorder mice results in decreased free carnitine levels and subsequently decrease in tissue and plasma LCAC accumulation but does not significantly affect clinically relevant outcome parameters (hypoglycemia, heart weight or liver weight) (Ranea-Robles et al., 2020). However, it should be noted that the C14:1-acylcarnitine levels were still higher in lcFAO disorder with OCTN2 haploinsufficiency when compared to wild type in heart, liver and plasma. Therefore, toxicity of acylcarnitines cannot be excluded.

In the present study, we have tested the effects of carnitine treatment on single VLCADD hiPSC-CMs and not on iCTRL-CMs. Whereas this approach allows a detailed study of the effects of a well-controlled dose of carnitine on the intrinsic LCAC concentrations, AP configuration and afterdepolarizations of the VLCADD hiPSC-CMs, it does not take into account the metabolic and structural complexity and heterogeneity of the intact myocardium or body. Therefore, caution is warranted when translating our *in vitro* results to the *in vivo* situation. Furthermore, although our VLCADD model has a human background thereby avoiding potential species differences, our study was limited to one patient-specific hiPSC line with *ACADVL* gene mutation. This provided us the opportunity to investigate the effects of carnitine on cardiomyocytes derived from this patient-specific hiPSC line in detail, but further studies are needed to test the carnitine effects on other VLCADD models. In the present study, we focused on LCAC concentrations and electrophysiology in hiPSC-CMs but VLCADD has a broad clinical spectrum (Ribas and Vargas, 2020; Wanders et al., 2020). In future studies, our VLCADD hiPSC line may also be differentiated to a liver and skeletal muscle cell fate (van der Wall et al., 2018; Corbett and Duncan, 2019) to assesses the effects of VLCADD and potential treatment in these cell types.

In conclusion, carnitine enhanced LCAC concentrations in hiPSC-CMs with *ACADVL* gene mutations and did not improve the electrophysiological abnormalities. This indicates that carnitine treatment is unlikely to be beneficial against cardiac arrhythmias in patients with *ACADVL* gene mutations.

## Data Availability Statement

The raw data supporting the conclusions of this article will be made available by the authors, without undue reservation.

## Author Contributions

SK, CB, and RH conceived and designed the study. KG generated the hiPSC lines. IM. maintained the hiPSC lines and differentiated them to cardiomyocytes. IM, SK, SF and JB dissociated cells and performed biochemical experiments. AV and VP performed electrophysiology measurements. SK and AV analyzed and interpreted the data and drafted the manuscript. LIJ, GV, RW, and FW contributed to scientific discussions. All authors critically revised the manuscript and gave final approval of the version to be published.

## Funding

KG was supported by the Free State of Saxony and the European Union EFRE (SAB project “PhänoKard”) and by the DFG (GU595/3-1, IRTG2251). CB was supported by the Dutch Heart Foundation (CVON PREDICT2 project), Netherlands Organization for Scientific Research (VICI fellowship, 016.150.610) and Fondation Leducq. RH was supported by a VIDI grant from ZonMw (no. 91715305) and a grant from the Velux Stiftung (no. 1063).

## Conflict of Interest

The authors declare that the research was conducted in the absence of any commercial or financial relationships that could be construed as a potential conflict of interest.

## Abbreviations

AP, action potential; APA, maximal action potential amplitude; APD_20_, action potential duration at 20% repolarization; APD_50_, action potential duration at 50% repolarization; APD_90_, action potential duration at 90% repolarization; Ca^2+^_i_, intracellular Ca^2+^; C_m_, cell membrane capacitance; DAD, delayed afterdepolarization; EAD, early afterdepolarization; hiPSC-CMs, human induced pluripotent stem cell derived cardiomyocytes; hiPSCs, human induced pluripotent stem cells; I_Ca,L_, L-type Ca^2+^ current; I_K1_, inward rectifying K^+^ current; I_to1_, transient outward K^+^ current; KO, knock out; LCAC, long-chain acylcarnitines; LCAD, long-chain acyl-CoA dehydrogenase; MDP, maximum diastolic potential; VLCAD, very long-chain acyl-CoA dehydrogenase; VLCADD, VLCAD deficiency; V_max_, maximum upstroke velocity.
